# Dual Chemotherapeutic Loading in Oxalate Transferrin-Conjugated Polymersomes Incorporated into Chitosan Hydrogels for Site-Specific Targeting of Melanoma Cells

**DOI:** 10.3390/ph17091177

**Published:** 2024-09-06

**Authors:** Mariana de C. Aranha, Luciana M. R. Alencar, Eliana B. Souto, Daniel T. Kamei, André M. Lopes

**Affiliations:** 1 Department of Biotechnology, Lorena School of Engineering, University of São Paulo (EEL/USP), Lorena 12602-810, Brazil; mariana.aranha@usp.br; 2 Laboratory of Biophysics and Nanosystems, Physics Department, Federal University of Maranhão, São Luís 65080-805, Brazil; 3 UCD School of Chemical and Bioprocess Engineering, University College Dublin, Belfield, Dublin 4, D04 V1W8 Dublin, Ireland; eliana.souto@ucd.ie; 4 Department of Bioengineering, University of California, Los Angeles, 420 Westwood Plaza, 5121 Engineering V, Los Angeles, CA 90095, USA

**Keywords:** polymersomes (Ps), PEG-PCL copolymers, chitosan hydrogels, doxorubicin, vemurafenib, oxalate transferrin (Tf)

## Abstract

In this work, we developed a smart drug delivery system composed of poly (ethylene glycol)-*block*-poly (ε-caprolactone) (PEG-PCL)-based polymersomes (Ps) loaded with doxorubicin (DOX) and vemurafenib (VEM). To enhance targeted delivery to malignant melanoma cells, these drug-loaded nanovesicles were conjugated to the oxalate transferrin variant (oxalate Tf) and incorporated into three-dimensional chitosan hydrogels. This innovative approach represents the first application of oxalate Tf for the precision delivery of drug-loaded polymersomes within a semi-solid dosage form based on chitosan hydrogels. These resulting semi-solids exhibited a sustained release profile for both encapsulated drugs. To evaluate their potency, we compared the cytotoxicity of native Tf-Ps with oxalate Tf-Ps. Notably, the oxalate Tf-Ps demonstrated a 3-fold decrease in cell viability against melanoma cells compared to normal cells and were 1.6-fold more potent than native Tf-Ps, indicating the greater potency of this nanoformulation. These findings suggest that dual-drug delivery using an oxalate-Tf-targeting ligand significantly enhances the drug delivery efficiency of Tf-conjugated nanovesicles and offers a promising strategy to overcome the challenge of multidrug resistance in melanoma therapy.

## 1. Introduction

Systemic chemotherapy can be effective in treating certain cancers, yet its efficacy is limited in treating melanoma, an aggressive form of skin cancer with a high risk of metastasizing [1,2,3]. Melanoma shows poor responsiveness to traditional systemic chemotherapy and radiotherapy, particularly in cases of large and poorly vascularized tumors. Surgical removal is the preferred therapeutic option when melanoma is resected, yet the risk of recurrence remains in the surrounding area or margins [4,5]. To overcome this challenge, a potential solution can involve directly administering chemotherapeutic agents to the affected area using controlled-release delivery systems. These modern approaches may involve using polymeric patches, scaffolds, and/or hydrogels placed within the injured (tumor) area to achieve targeted and sustained drug delivery. Chitosan hydrogels (CHs) offer a unique capacity for localized and sustained release of medications, ensuring high concentrations of drugs specifically within the tumor region for an extended duration, all the while minimizing widespread circulation. A significant achievement in this context involves employing chitosan-based hydrogels with entrapped drugs, providing an efficient method for administering antitumoral drugs for melanoma treatment [6]. This innovative strategy enhances the effectiveness of treatment, reduces toxicity, minimizes the risk of drug resistance, and decreases the number of chemotherapy sessions. As a result, it promotes patient compliance with treatment protocols and improves overall quality of life [7,8,9,10].

Numerous studies exploit the integration of therapeutic medications (either in their free form or encapsulated within nanostructures) into three-dimensional hydrogels made of chitosan, with successful outcomes both *in vitro* and *in vivo* [11,12,13,14,15,16]. CHs are well known for their low toxicity, biocompatibility, and mucoadhesiveness. Furthermore, CHs have been extensively researched as a matrix for sustained drug delivery [17]. While incorporating unencapsulated drugs in CHs has resulted in important technological advances, their efficacy remains hindered due to the potential side effects arising from the intrinsic high toxicity of chemotherapeutic and antineoplastic drugs [8,18]. Encapsulating such highly toxic drugs into nanovesicles (i.e., polymersomes—Ps) before embedding them in CHs has reduced toxicity.

Nonetheless, the therapeutic effectiveness of these nanovesicles remains limited due to their low specificity, which leads only to a portion of the drugs arriving at the desired cancer cells [8,18]. Luckily, the issue of low specificity between Ps and cancer cells can be overcome by harnessing the efficient cellular uptake mechanism facilitated by protein transferrin (Tf). This protein, known for its cancer cell-targeting properties, has been used in diverse delivery systems due to the frequent overexpression of the transferrin receptor (TfR) in numerous cancer cell types [19,20], including in A431 [21,22] and A375 cell lines [23,24].

While the attachment of native (unmodified) Tf to therapeutic agents enhances targeted uptake by tumor cells, there are notable limitations to its effectiveness as a drug carrier. The short time period associated with the passage of holo-Tf (iron-bound Tf) through the cell, typically only 5 to 10 min, largely contributes to this phenomenon [25]. Upon binding to the TfR, holo-Tf undergoes receptor-mediated endocytosis, releasing its iron payload. The resulting apo-Tf (iron-free Tf) is subsequently recycled back to the cell surface, where it quickly dissociates from the TfR. Since the process of Tf rebinding iron is inefficient, recycled Tf is typically assumed to be unable to reacquire iron [26,27]. As a result, the drug delivery vehicle conjugated to Tf only has a short time in which to deliver its payload. We have achieved a significant breakthrough by successfully generating a Tf variant with oxalate replacing the carbonate anion. This innovative modification has led to a remarkable increase in cellular association compared to native Tf. As a result, the delivery of a conjugated drug has been significantly enhanced, opening up exciting possibilities for targeted drug delivery [28,29,30].

Our ongoing study expands our previous research, aiming to maximize the potential of poly (ethylene glycol)-*block*-poly (ε-caprolactone) (PEG-PCL) nanovesicles for the simultaneous encapsulation of two drugs, specifically doxorubicin (DOX) and vemurafenib (VEM). In our recently published work, we examined PEG_y_-PCL_x_ copolymers. Significantly, our findings indicated that Ps with smaller PEG-PCL chains, such as PEG_45_-PCL_44_, exhibited the highest drug release rate, followed by PEG_114_-PCL_98_ and PEG_114_-PCL_114_. Moreover, our results unveiled that PEG-PCL Ps loaded with DOX and VEM exhibited an improved drug release pattern in acidic conditions (pH 5.0 at 37 °C), resembling the environment of cancer cells, in contrast to the release observed under physiological conditions (pH 7.4 at 37 °C) [31].

In the present study, we evaluated the potential enhanced efficacy of oxalate Tf-conjugated DOX and VEM-loaded PEG-PCL Ps incorporated within three-dimensional CHs. The main aim of this study was to determine whether the utilization of oxalate Tf, rather than native Tf, would result in the improved cellular association and performance of the Ps. Based on current understanding, this study is the first to demonstrate the use of oxalate Tf for enhancing targeted drug delivery of Ps incorporated within CHs to melanoma cancer cells.

## 2. Results and Discussion

### 2.1. Stability and Characterization of Polymersomes

**Figure 1:**
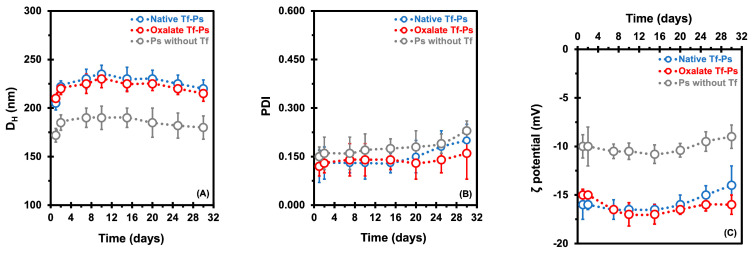
Measurement of the hydrodynamic diameter (D_H_, in nm) (**A**), polydispersity index (PDI) (**B**), and ζ potential (in mV) (**C**) was carried out to characterize the PEG-PCL-based polymersomes (Ps). Ps were evaluated *in vitro* at 37 °C for 1 to 30 days in PBS buffer (pH 7.4). Dynamic light scattering analyses were carried out at 37 °C. The error bars depicted correspond to the 95% confidence intervals calculated based on three independent measurements. It is worth noting that in certain cases, the error bars are smaller than the markers themselves.

The PEG-PCL-based Ps were characterized regarding their *in vitro* stability at 37 °C throughout 1 to 30 days. This involved evaluating changes in size (D_H_), polydispersity index (PDI), and ζ potential, as depicted in Figure 1A,C. Tf is a protein with a molecular weight of 78 kDa and dimensions of approximately 9.5 nm × 6 nm × 5 nm [32]. The average D_H_ values of the native and oxalate Tf-Ps were determined to be 225 ± 7.0 nm and 221 ± 11 nm, respectively, representing an approximate increase of ~39 nm compared to the diameter of the Ps without Tf (184 ± 9.0 nm), suggesting that a Tf monolayer was conjugated to the Ps (Figure 1A). During the 30 days at 37 °C, the D_H_ values of the native Tf-Ps ranged from 205 to 220 nm, while those of the oxalate Tf-Ps ranged from 210 to 215 nm. In contrast, the Ps without Tf control group exhibited D_H_ values of 172 to 180 nm. Although the nanovesicles displayed good stability under the examined conditions, a slight increase in the Ps’ size over time was observed. In a recent investigation [31], we attributed this phenomenon to a dual effect involving the hydration/dehydration dynamics of the PEG corona and the degradation of Ps over time. Initially, hydration of the Ps nanovesicles occurred, potentially due to water absorption and swelling, leading to a slight increase in D_H_ attributed to the hydrophilic PEG chains on the P surface. Conversely, a declining trend in nanovesicle diameter emerged in the later stages of analysis, suggesting potential early signs of copolymer chain degradation. It is noteworthy that the degradation process of polymers may accelerate at elevated temperatures (>37 °C) [31,33,34]. In parallel, transmission electron microscopy (TEM) analyses were performed to confirm the self-assembly into nanovesicles (Figure S2, Supplementary Materials).

Another crucial parameter for assessing the stability of nanovesicles is the PDI values. The PDI values of our Ps ranged from 0.120 to 0.230 at 37 °C over 30 days (Figure 1B), indicating the presence of monodisperse systems characterized by homogeneous populations of nanovesicles. Tf depicts a slightly negative charge at physiological pH (p*I* = 5.5 to 6.1) [35]. As anticipated, the average ζ potentials of Ps with ligands (−15.8 ± 1.0 mV for native Tf-Ps and −16.1 ± 1.1 mV for oxalate Tf-Ps) were more negative compared to Ps without Tf (−10.0 ± 0.7 mV), as depicted in Figure 1C. While there is a general guideline for maintaining the stability of nanostructures (around ± 30 mV in absolute value for ζ potential), the stability of PEG-PCL-based Ps nanovesicles is predominantly influenced by the steric effect arising from the dense PEG shell, rather than a specific surface charge.

### 2.2. Co-Encapsulation of Drugs in Polymersomes

The successful co-encapsulation of DOX and VEM by Ps nanovesicles is shown in Table 1, showcasing the effectiveness of each nanovesicle. This is evident from the *DL* and *EE* values, indicating the efficient encapsulation of both drugs. For DOX, the *DL* values ranged from 10.0% for the native Tf-Ps and 11.6% for the oxalate Tf-Ps to 12.1% for the Ps without Tf. Similarly, VEM exhibited *DL* values ranging from 16.3% for the native Tf-Ps to 18.1% for the oxalate Tf-Ps, with an intermediate value of 17.3% for the Ps without Tf. The *EE* values for the Ps without Tf for DOX and VEM were 35.0 and 42.2%, respectively. The native Tf-Ps showed efficiencies of 32.2% for DOX and 40.4% for VEM, while the oxalate Tf-Ps demonstrated 33.8% for DOX and 39.5% for VEM (Table 1).

Furthermore, although not statistically significant, VEM exhibited higher values of *DL* and *EE* compared to DOX (*DL*_VEM_ > *DL*_DOX_ and *EE*_VEM_ > *EE*_DOX_). This phenomenon is a result of the inherent hydrophobicity of VEM, which promotes its interaction with the hydrophobic bilayer of the nanovesicles due to the energy penalty associated with drug–water interactions. On the other hand, DOX encapsulation primarily involves its entrapment in the interior aqueous phase within the nanovesicles, with no distinct affinity between copolymers/nanovesicles and DOX. As a result, the primary factor influencing *EE* is the initial concentration of the drug in the hydration solution. This might clarify the higher observed values for VEM in terms of *DL* and *EE* [31].

Our findings align with existing literature reports on nanovesicles. For instance, Colley et al. [36] described pH-sensitive Ps for encapsulating DOX and paclitaxel (PTX), achieving *EE* values of 37.1% (DOX) and 42.7% (PTX). In another work, Iatrou et al. [37] examined nanovesicles, reporting DOX and PTX having *DL* of 19.5 and 33%, and *EE* of 13 and 25%, respectively.

### 2.3. Studies of Degradation and Swelling of Chitosan Hydrogels

**Figure 2:**
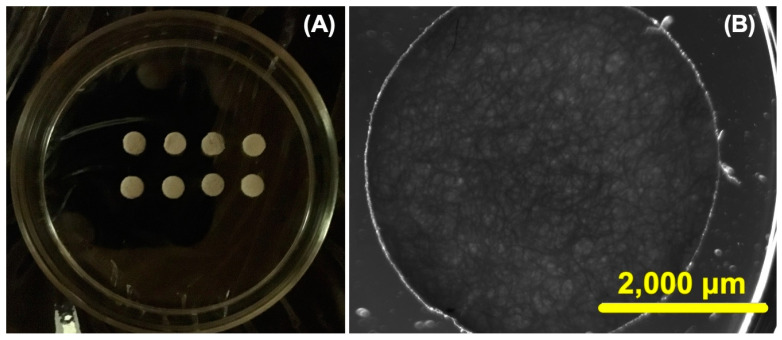
The cylindrical chitosan hydrogels (CHs) are visible under ordinary light, as depicted in the following images: (**A**) the CHs were cut into circular samples and (**B**) light and dark field microscopy was employed to show the CHs at 7 days (scale bar is 2000 μm and the objective was set at 2×).

Initially, we carefully designed and constructed CHs with dimensions of 4 mm in diameter and 1 mm in thickness (Figure 2A). As shown in Figure 2B, the complex three-dimensional polymeric network within the CHs becomes apparent under microscopy one week after their fabrication. Our objective was not to conduct in-depth studies on the physicochemical characterization of CHs (such as Fourier transform infrared spectroscopy, nuclear magnetic resonance, and scanning electron microscopy), as these hydrogels have already been extensively characterized and modified in the literature; for more details about the physicochemical characterization of CHs, we recommend the following works [38,39,40]. Instead, our primary focus was simply to obtain the CHs for incorporation into the nanoformulations (native and oxalate Tf-Ps), which were the main focus of our study.

**Figure 3:**
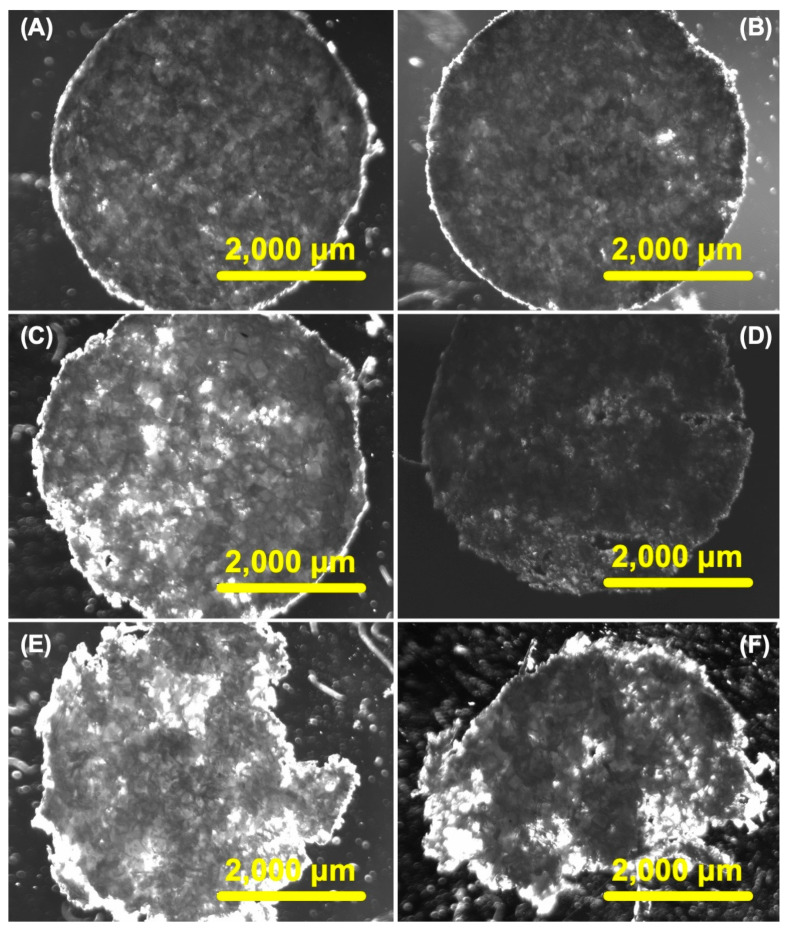
*In vitro* degradation of chitosan hydrogels (CHs) over different periods while being stored in 10 mM PBS buffer (pH 7.4). The time points examined were 30 (**A**), 60 (**B**), 90 (**C**), 120 (**D**), 150 (**E**), and 180 days (**F**). Light and dark field microscopy was employed to visualize the CHs, with scale bars of 2000 μm and the objective set at 2×.

The hydrolytic degradation of the CHs was systematically examined over time in physiological conditions (i.e., PBS pH 7.4 at 37 °C) for durations of 30, 60, 90, 120, 150, and 180 days. To elucidate the degradation process, we obtained bright field microscopy images of the porous CHs (Figure 3A–F). The images vividly demonstrated substantial degradation by the 120-day mark (Figure 3D), and this degradation became even more pronounced at 150 and 180 days (Figure 3E and Figure 3F, respectively). These images exhibited distinct dark regions on these days, indicative of the depletion of polymer material. In contrast, minimal changes were discernible in the bright field microscopy images at days 30 and 60 (Figure 3A and Figure 3B, respectively). To further explore the potential that degradation has on drug diffusion, we carried out swelling and release studies.

**Figure 4:**
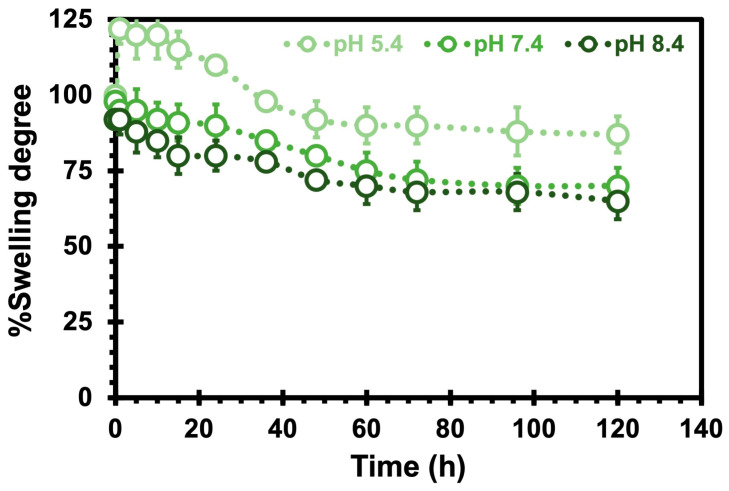
The swelling degree of chitosan hydrogels as a function of hours at 37 °C at different pH values. The error bars depicted correspond to the 95% confidence intervals calculated based on three independent measurements. It is worth noting that in certain cases, the error bars are smaller than the markers themselves.

To investigate the drug-release characteristics of the CHs, we measured the swelling ratio over time at various pH levels (Figure 4). At pH 5.4, the CHs initially underwent swelling for the first 10 h, followed by a subsequent shrinking phase over the next 120 h. However, at pH 7.4 and 8.4, the CHs consistently exhibited a shrinking behavior throughout the entire duration. These findings suggest that when the surrounding pH increases, the hydrogel experiences significant shrinkage, effectively expelling a large amount of water from its network. Similar observations were made by Wei et al. [41] in their study on chitosan (CS)/poly (vinyl alcohol) (PVA) hydrogels, where they demonstrated that the release rate of aspirin was pH-dependent with a faster release observed at higher pH values (i.e., 8.4 and 7.4 compared to 4.0 and 5.5). Consistent with findings reported in the literature [42], hydrogel degradation correlates with decreasing swelling ratios. This significant relationship reinforces the swelling data and provides valuable insights into the hydrogel’s suitability as a nanocarrier. Considering the potential future application of these hydrogels in filling injured or tumor areas of specific sizes, comprehending their swelling characteristic becomes crucial to guarantee safe and secure placement. Although the results obtained at higher pH levels are not directly relevant to our study, these outcomes are highly valuable to explore future applications beyond oncology and related fields.

### 2.4. Studies on Drug Release from Chitosan Hydrogels

**Figure 5:**
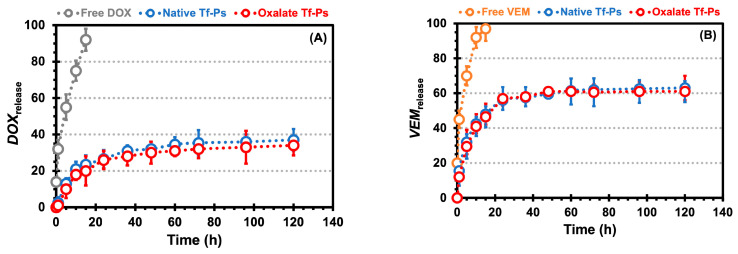
The doxorubicin (DOX, **A**) and vemurafenib (VEM, **B**) release profiles from the chitosan hydrogels (CHs) in PBS (pH 7.4) over time. Free drug corresponds to the drug encapsulated directly in CHs, while the Tf-Ps correspond to the drug encapsulated in Tf-Ps that were then incorporated in CHs. The error bars depicted correspond to the 95% confidence intervals calculated based on three independent measurements. It is worth noting that in certain cases, the error bars are smaller than the markers themselves.

Evaluating the performance of nanovesicles is pivotal, particularly through *in vitro* drug delivery assessments. It is commonly noted that a robust connection exists between *in vitro* conditions and the *in vivo* behavior of nanocarriers [43]. Figure 5A,B depict the time-dependent release patterns of DOX and VEM from CHs, alongside the release profiles of DOX and VEM from CHs incorporating native and oxalate Tf-Ps, in PBS (pH 7.4) at 37 °C. The release of drugs from biomaterials encompasses a combined diffusion and erosion mechanism, occurring either sequentially or concurrently [44]. In these performed experiments, it was noted that the release of drugs from the CHs was faster compared to that of the drugs encapsulated in native and oxalate Tf-Ps incorporated in CHs. Notably, the drugs directly encapsulated in CHs exhibited a burst effect by 15 h. The observed release profile is a result of the predominant mechanism being straightforward diffusion within the hydrogel matrix.

Additionally, the release of VEM from the CHs (i.e., 45.0% at 1 h and 97.0% at 15 h) exhibited a faster rate than the release of DOX from the CHs (i.e., 32.0% at 1 h and 92.0% at 15 h). This trend was also consistent for the release of VEM from the Tf-Ps incorporated in the CHs (i.e., 15.5% at 1 h and 48% at 15 h for native and 12% at 1 h and 46.5% at 15 h for oxalate) when compared to the release of DOX from the Tf-Ps incorporated in the CHs (i.e., 2.5% at 1 h and 23.5% at 15 h for native and 1.0% at 1 h and 20.0% at 15 h for oxalate), as shown by comparing Figure 5B *versus* Figure 5A. Several hypotheses can be proposed to explain this observation. One possibility is that with rising temperature, the PEG chains within the nanovesicles undergo dehydration, resulting in a more hydrophobic environment that promotes faster release. This well-documented phenomenon causes partial dehydration and several significant changes, including the disruption of structured hydration water, the weakening of repulsive hydration forces, intra-/inter-vesicular overlapping, and decreased hydrophilicity of the PEG corona. Consequently, components encapsulated within the vesicular structure are released at an accelerated rate [45,46,47,48]. More specifically, Qu et al. [49] investigated the release rates of folic acid linked to the thermo-responsive corona of polymeric micelles composed of a copolymer. When achieved values below the lower critical solution temperature (around 25 °C), folic acid was effectively retained within the polymeric micelle’s core, as its diffusion through the hydrophilic corona was restricted. Nevertheless, as temperatures exceeded 40 °C (surpassing the lower critical solution temperature), the corona area transitioned towards increased hydrophobicity, enabling the hydrophobic drug to easily infiltrate this domain. As the corona is near the bulk aqueous solution, the drug could distribute into the aqueous surroundings and migrate into the surrounding bulk water, driven by differences in solute concentration in the external aqueous phase. Our observations support this hypothesis, as higher temperatures seem to facilitate drug penetration through the PEG-PCL-based Ps nanovesicle corona. The accelerated release of VEM, situated in the bilayer and in proximity to the outer layer (i.e., PEG corona), may be attributed to this phenomenon. Additionally, our research group posits that various diffusion-related factors played a role in the increased release rate at elevated temperatures [50,51]. Particularly, higher temperatures lead to increased thermal motion, affecting both the stability of the nanovesicles and the mobility of the drugs, thereby facilitating drug diffusion. Additionally, as temperature rises, the reduced viscosity can further enhance the diffusion process [31].

In spite of the various distinctive features displayed by biocompatible and biodegradable nanovesicles, attaining exact management of drug release remains a noteworthy obstacle. The release kinetics of encapsulated drugs in nanovesicles are primarily influenced by the polymer’s structure and properties, including the ratio of hydrophilic to hydrophobic blocks, molecular weight, and the hydrolytic degradation of the hydrophobic chains [52]. Overall, our findings highlight the potential of PEG-PCL-based Ps as versatile nanovesicles for temperature- and pH-responsive drug release [31]. Moreover, these nanocarriers offer controlled and extended drug release for up to 5 days (for DOX at 5 days: 37.0 and 34.0% for native and oxalate, respectively, and for VEM at 5 days: 63.0 and 61.0% for native and oxalate, respectively), thereby presenting promising opportunities for targeted and sustained therapeutic interventions.

### 2.5. In Vitro Cytotoxicity Evaluation

**Figure 6:**
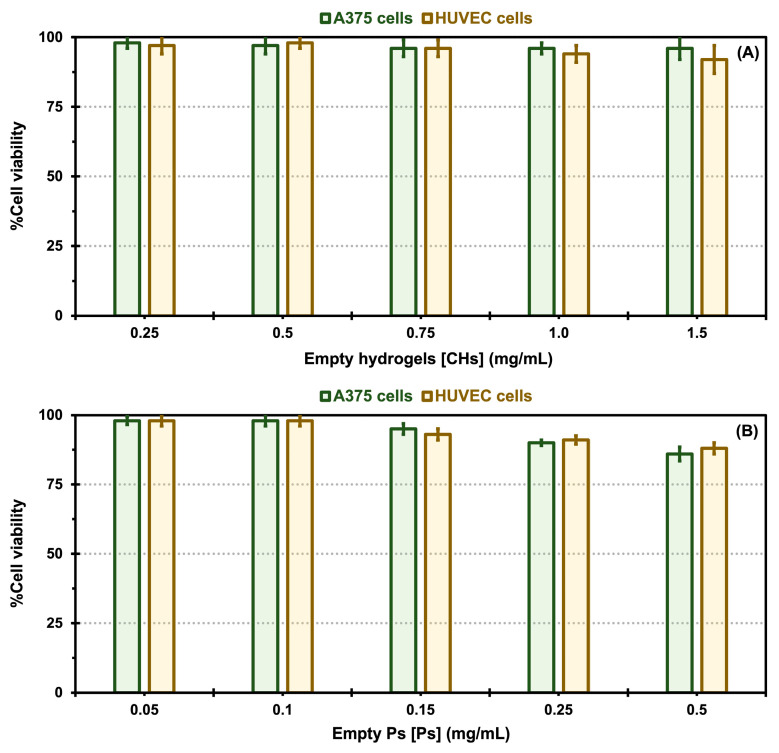

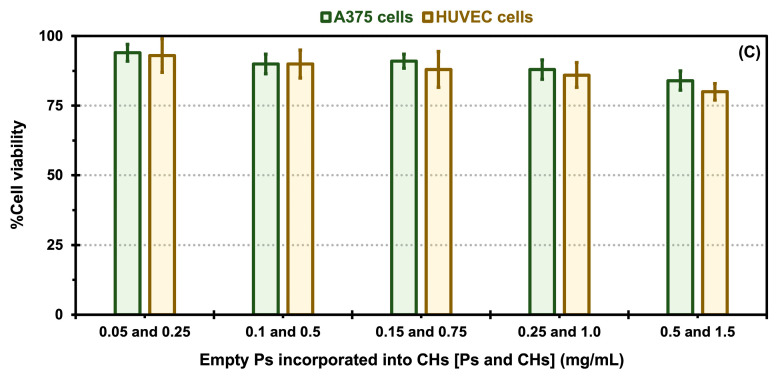
*In vitro* cytotoxicity comparisons for control samples: empty chitosan hydrogels (CHs, **A**), empty polymersomes (Ps, **B**), and empty Ps incorporated into CHs (**C**) exposed to A375 melanoma cells and HUVEC cells. Error bars depicted correspond to 95% confidence intervals calculated based on three independent measurements.

**Figure 7:**
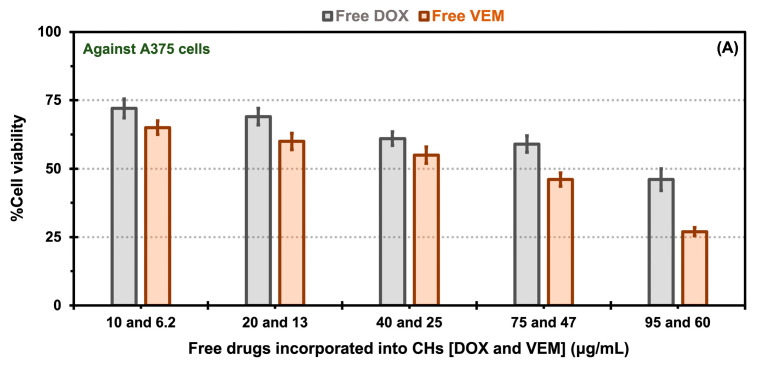

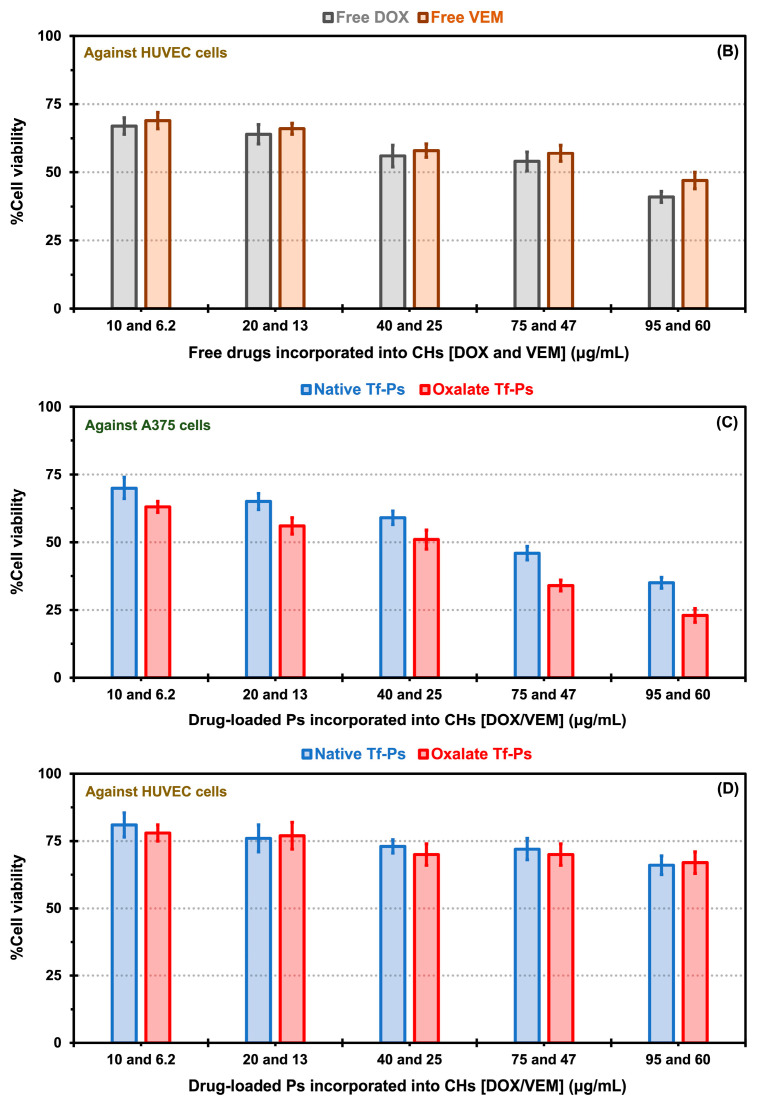
*In vitro* cytotoxicity comparisons for free drugs [doxorubicin (DOX) and vemurafenib (VEM)] incorporated into chitosan hydrogels (CHs) exposed to A375 melanoma cells (**A**) and HUVEC cells (**B**), as well as native and oxalate transferrin (Tf) conjugated to the PEG-PCL-based polymersomes (Ps) incorporated into CHs and exposed to A375 melanoma cells (**C**), and HUVEC cells (**D**). The error bars depicted correspond to the 95% confidence intervals calculated based on three independent measurements.

A cytotoxicity analysis was carried out to assess the impact of different nanoformulations, including empty CHs and Ps, and empty Ps (without drug encapsulation) incorporated into CHs, free DOX encapsulated in CHs, and free VEM encapsulated in CHs, as well as native and oxalate Tf-Ps encapsulated in CHs, on the proliferation of malignant A375 melanoma cells and normal HUVECs, which are reported to express Tf receptors [23,24,53]. The results of the MTS assay are presented in Figure 6 and Figure 7. The results demonstrate that different concentrations of all control samples exhibited excellent biocompatibility and did not exhibit cytotoxic effects on both cell types, with cell viability values between 96 to 98% (for A375 cells) and 92 and 97% (for HUVEC cells) for the empty CHs, 86 to 98% (for A375 cells) and 88 and 98% (for HUVEC cells) for the empty Ps, and 84 to 94% (for A375 cells) and 80 and 93% (for HUVEC cells) for the empty Ps incorporated into CHs, as demonstrated in Figure 6A Figure 6B and Figure 6C, respectively.

In the experiments involving free drug incorporated into CHs, A375 cell viability displayed a decrease dependent on concentration for both free drugs. The viability of A375 cells in the presence of free DOX ranged from 46 to 72% for DOX concentrations between 95 and 10 μg/mL, respectively. Similarly, for free VEM, the viability ranged from 35 to 65% for VEM concentrations between 60 and 6.2 μg/mL, respectively (Figure 7A). For HUVECs, the cell viability for free DOX showed values of 41 to 67% for DOX concentrations between 95 and 10 μg/mL, respectively, and for free VEM of 47 to 69% for VEM concentrations between 60 and 6.2 μg/mL, respectively (Figure 7B). Notably, free VEM exhibited greater toxicity towards A375 cells (Figure 7A) than HUVECS (Figure 7B), which could be attributed to its specific effects on this type of cells. The BRAF^V600E^ mutation is common in melanoma cells, resulting in constitutive activation of the BRAF protein kinase. VEM is a targeted therapy that specifically inhibits the activity of the mutant BRAF protein kinase. By blocking the activity of BRAF^V600E^, VEM effectively suppresses the signaling pathways that promote the growth and survival of BRAF^V600E^ melanoma cells [54]. This high specificity of the free drug in killing more A375 cells than HUVECS may explain the effect observed in our results (e.g., the 60 μg/mL of free VEM was 1.7-fold more toxic for A375 cells than for HUVECS).

There was a notable decrease in cellular viability for both drug formulations, whether encapsulated in native or oxalate Tf-Ps and incorporated into CHs, across various concentrations, in contrast to the cell viability observed with free drug incorporation into CHs. The increased cell viability associated with free drugs and the reduced cell viability with native and oxalate Tf-Ps may be attributed to the amplified cellular uptake of the Tf-conjugated nanovesicles. This is particularly interesting because, *in vivo*, another important factor contributing to these results is the enhanced permeability and retention (EPR) effect. This effect naturally retains drugs (or Ps) at the tumor site. Nanostructures passively accumulate in solid tumors that have immature and leaky vasculature, with wider fenestrations than normal blood vessels, as described by Singh [55] for the dual loading of DOX and celecoxib in liposomes tested against a squamous carcinoma cell line. Moreover, when comparing native *versus* oxalate Tf-Ps, A375 cell viability at DOX and VEM concentration of 95 and 60 μg/mL showed values of 35 *versus* 23% (Figure 7C). Similar findings have been reported in the literature, where PLGA nanoparticles decorated with oxalate Tf demonstrated improved cellular association, and therefore improved drug carrier efficacy, compared to native Tf nanoparticles [29]. The obtained results validate the improved interaction of oxalate Tf-Ps with cells, leading to enhanced effectiveness of therapeutic agents (i.e., cell viability of 23% with oxalate Tf-Ps *versus* 35% with native Tf-Ps) when incorporated into CHs. Furthermore, combining two drugs, nano size, drug protection, and targeted release contributed to the highest toxicity in cancer cells observed with the oxalate Tf-Ps at a specific concentration. Our *in vitro* cytotoxicity experiments quantitatively revealed an approximate 1.5-fold reduction in cell viability values when comparing native and oxalate Tf-Ps in BRAF^V600E^ melanoma cells. This was achieved without further decreasing HUVEC viability, as the same DOX and VEM concentrations (95 and 60 μg/mL) yielded similar viability percentages (66 *versus* 67% for native *versus* oxalate Tf-Ps). These viability percentages were 1.9- and 3-fold higher than those obtained with A375 cells (Figure 7D). The strong toxic effect of these nanoformulations on A375 cells, as previously mentioned, is linked to the overexpression of TfR by tumor cells. This effect becomes even more apparent when comparing the expression levels of these receptors among the evaluated cell types. TfR expression is significantly higher in A375 cells than in HUVECs, as demonstrated by Western Blot assays in Figure S3 (Supplementary Materials).

One notable advantage of dual-drug nanocarriers, such as our nanovesicles, is the synergistic effect achieved by combining two drugs within the system. The literature describes some examples involving dual-drug encapsulation into polymersomes (e.g., DOX and gemcitabine, DOX and PTX, oxaliplatin and rapamycin, curcumin and methotrexate) [56,57,58,59]. Each drug possesses unique mechanisms for destroying cancer cells, and when combined, these mechanisms complement each other, leading to enhanced effectiveness in targeting and eliminating cancer cells [60]. By harnessing the advantages of dual-drug delivery, our nanovesicles demonstrate the potential to increase toxicity toward cancer cells and optimize therapeutic outcomes. In chemotherapy, various nanostructures composed of safe materials have been developed as delivery systems for VEM. However, it is commonly observed that the effectiveness of VEM treatment diminishes within approximately seven months [61,62]. This observation suggests that tumors develop resistance to VEM over the long term. The exact mechanism underlying the development of acquired therapeutic resistance in melanoma remains unclear. It is uncertain whether resistance emerges through an evolutionary process within the tumor or if pre-existing resistant clones are selected during therapy, highlighting the genetic heterogeneity of tumors [63,64].

For this reason, the dual-drug delivery from our nanovesicles presents a promising solution to overcome this obstacle and prevent the development of multidrug resistance (MDR) during treatment. To ensure suitability for clinical use, CH-Ps can be produced without pyrogens, following the guidelines of good manufacturing practices. Our findings demonstrate that the controlled release characteristic of CH-Ps allows for continuous and prolonged drug delivery. This unique feature offers several advantages, including preventing variations in the concentration of drugs in the bloodstream over time, as well as the potential for achieving maximum pharmacological efficacy with minimal drug dosage. Our observations imply the potential for these platforms to act as an alternative to free drugs, facilitating the controlled and extended release of chemotherapeutic agents from CHs without compromising their anticancer potential. Furthermore, these platforms show promise and motivation for further development, aiming to mitigate the adverse reactions of free drugs.

## 3. Materials and Methods

### 3.1. Materials

The copolymer composed of poly (ethylene glycol) methyl ether-*block*-poly (ε-caprolactone) (PEG_45_-PCL_44_) was acquired from Polymer Source Inc. (Montreal, Quebec, Canada), with a molecular weight of 7000 g/mol and acid termination. The copolymer was carefully stored to protect it from light and moisture until further use. The drugs (vemurafenib and doxorubicin hydrochloride) were acquired from Sigma-Aldrich (Saint Louis, MO, USA) and LC Laboratories (Boston, MA, USA), respectively. Similar storage precautions were taken for these materials. The chitosan from shrimp shells with ≥75% deacetylated with medium molecular weight was acquired from Sigma-Aldrich. Ultrapure water (Millipore Milli-Q system, Bedford, MA, USA) was used to prepare the solutions of all components under *in vitro* physiological conditions [i.e., 10 mM phosphate-buffered saline (PBS) at pH 7.4]. All reagents employed in this work were of analytical grade and required no further modification before use.

### 3.2. Polymersome Preparation

The nanovesicles were prepared using a method adapted from D’Angelo et al. [31]. Initially, a predetermined quantity of the PEG_45_-PCL_44_ copolymer was dissolved in CHCl_3_ at 0.06% (*w/v*) in a glass volumetric flask. The CHCl_3_ was evaporated using an IKA RV 10 evaporator (IKA GmbH, Staufen im Breisgau, Germany) and dried under vacuum conditions (0.35 mbar) for 1 h until CHCl_3_ removal. The hydration of the thin polymeric film was performed with buffer solution (i.e., 10 mM PBS at pH 7.4) while being stirred magnetically at 1200 rpm (IKA RH basic, IKA GmbH, Staufen im Breisgau, Germany) for 40 h at room temperature. In some cases, DOX was introduced to the PBS solution at 3% (*w/v*) to facilitate its incorporation into the nanovesicles during hydration. For the encapsulation of VEM, a separate solution of the drug was prepared by dissolving VEM in DMSO at 3% (*w/v*). This VEM solution was then added to the DOX-Ps suspension at a volume ratio of 12% (*v/v*) through sonication. Sonication was performed using a Qsonica^®^ ultrasound device (Columbiana County, OH, USA) at 30 °C and 50 W for 45 min. This step not only facilitated the incorporation of VEM but also contributed to reducing the polydispersity of the nanovesicles. Following the sonication step, the nanovesicles were transferred into dialysis bags with a molecular weight cut-off of approximately 1 MDa and subjected to five buffer solution exchanges as described above at 8 °C. This process effectively removed any residual DMSO and excess-free drugs. Subsequently, the samples went through three rounds of filtration using a PVDF membrane with a pore size of 220 nm. Finally, the Ps were subjected to lyophilization and preservation at −80 °C to maintain their stability.

The conjugation of Tf onto the Ps, along with the generation of oxalate Tf and iron loading of Tf, followed the methodology outlined by our research group (Lopes et al. [28]). For more detailed information, please refer to the Supplementary Materials (Section S1).

### 3.3. Chitosan Hydrogels Preparation and Incorporation of the Polymersomes

In summary, a chitosan solution (2.5%) was created by dissolving 0.025 mM chitosan in 22.0 mL of acetic acid (0.025 mM) through continuous magnetic stirring at 600 rpm overnight, following the procedure outlined by Wande et al. [65]. The stirring continued until a clear chitosan solution formed. Subsequently, 2.0 mL of aqueous iodoacetic acid (0.215 mM) was carefully added dropwise to the chilled chitosan solution while maintaining a temperature of 0 °C. The mixture underwent magnetic stirring (600 rpm) for 3 h. Afterward, the reaction mixture underwent filtration using a 220 nm pore size membrane. The resulting paste was then transferred to a 48-well tissue culture plate, and the resulting CHs were dried in an oven at 55 °C overnight. Following drying, the samples underwent sterilization in 70% EtOH for 45 min and were rinsed three times with sterile PBS buffer (10 mM, pH 7.4). The incorporation of nanovesicles into the CHs occurred by hydrating them with 500 μL of a solution containing various concentrations of both drugs (either as combined free drugs or in Tf-Ps), ranging from 10 to 95 μg/mL for DOX and 6.2 to 60 μg/mL for VEM, respectively, in microtubes. In our results, the combination of the drugs was represented as DOX and VEM at 10 and 6.2, 20 and 13, 40 and 25, 75 and 47, and 95 and 60 μg/mL, respectively. The CHs were exposed to these solutions on a rotary shaker (100 rpm) at 25 °C overnight. After this step, the samples underwent three washes with PBS buffer to eliminate any free drugs and Ps from the CHs’ surface.

### 3.4. Swelling and Degradation of Chitosan Hydrogels

The stability of CHs was assessed by evaluating their *in vitro* swelling and degradation over time. To assess the swelling behavior of the CHs, we performed measurements under simulated physiological conditions at 37 °C. Briefly, freshly prepared CH samples (in their wet state) were weighed accurately. The CHs were stored in a 48-well plate containing 1.0 mL of PBS buffer solution with pH values of 5.4, 7.4, and 8.4. The plate was then placed in a humidified environment at 37 °C with 5% CO_2_, fully immersed in PBS buffer. After specific intervals (0 to 120 h), the samples were removed from the buffer solution, gently wiped with filter paper to eliminate any excess surface solution, and reweighed. The samples were then returned to their respective containers. The swelling degree (SD) of the CHs over time was calculated using Equation (1), as described by Wei et al. [41].

(1) $$\mathrm{SD}\;\left(\%\right)=\left(\frac{W_{t}\;\mathrm{is}\mathrm{the}\;\mathrm{weight}\;\mathrm{of}\;\mathrm{hydrogel}\;\mathrm{at}\;\mathrm{various}\;\mathrm{swelling}\;\mathrm{times}}{W_{0}\;\mathrm{is}\;\mathrm{the}\;\mathrm{weight}\;\mathrm{of}\;\mathrm{the}\;\mathrm{original}\;\mathrm{hydrogel}}\right)\times 100$$

To evaluate the degradation of the CHs, multiple images were captured at various time intervals (1, 30, 60, and 90 days) using an EVOS FL Imaging System for bright-field microscopy (Thermo Fisher Scientific, Waltham, MA, USA) in PBS buffer solution.

### 3.5. Dimensional Stability and Characterization of Polymersomes

The size (hydrodynamic diameter—D_H_) and polydispersity index (PDI) of the nanovesicles were determined using dynamic light scattering (DLS). Measurements were conducted at 25 °C with the Malvern Nano-ZS 90 device (Malvern Instruments in Worcestershire, UK). Nanovesicles were appropriately diluted by a factor of 1000 when necessary. Additionally, the surface charge of the nanovesicles [zeta (ζ) potential] was measured using the same device. The reported results represent the average of three independent assays. Lyophilized samples were incubated in small vials with 3.0 mL of PBS buffer (pH 7.4) at 37 °C, and the nanovesicles were monitored over 1 to 30 days. Following each period of time, the nanovesicles were evaluated by measuring D_H_, PDI, and ζ potential, as described above.

### 3.6. Determination of Encapsulation Parameters

To assess the parameters of drug loading and efficiency in encapsulation (DL and EE, respectively), we followed a procedure adapted from D’Angelo et al. [31]. Here is a brief overview of the steps involved. We added 400 μL of samples containing the drug-Ps into pre-weighed microtubes. Then, these samples were subjected to overnight freezing at approximately −80 °C for about 10 h. After freezing, the samples underwent overnight lyophilization using a FreeZone 6lt lyophilizer (Labconco Corp., MO, USA) with a condenser surface temperature of −50 °C and a pressure of 350 mTorr. This process lasted for around 10 h. Following lyophilization, we weighed the samples to determine their dry masses. Next, we dissolved the drug-Ps nanovesicles in 400 μL of DMSO. The drugs trapped in the nanovesicles were then estimated using the method defined below. Using the data obtained, we calculated the parameters according to the Equations (2) and (3):

(2) $$\mathrm{DL}\;\left(\%\right)=\left(\frac{\mathrm{Weight}\;\mathrm{of}\;\mathrm{drugs}\;(\mathrm{DOX}\;\mathrm{and}\;\mathrm{VEM})\;\mathrm{entrapped}\;\mathrm{in}\;\mathrm{Ps}}{\mathrm{Total}\;\mathrm{weight}\;\mathrm{of}\;\mathrm{drug-Ps}}\right)\times 100$$

(3) $$\mathrm{EE}\;\left(\%\right)=\left(\frac{\mathrm{Weight}\;\mathrm{of}\;\mathrm{drugs}\;(\mathrm{DOX}\;\mathrm{and}\;\mathrm{VEM})\;\mathrm{entrapped}\;\mathrm{in}\;\mathrm{Ps}}{\mathrm{Weight}\;\mathrm{of}\;\mathrm{drug}\;\mathrm{initially}\;\mathrm{added}\;\mathrm{in}\;\mathrm{formulation}}\right)\times 100$$

### 3.7. Drug Quantification

The concentrations of the drugs were determined with a Microplate Spectrophotometer from Thermo Fisher Scientific (Waltham, MA, USA). The absorbance measurements were taken at 305 and 490 nm for VEM and DOX, respectively. To ensure accuracy, ultrapure water (Millipore Milli-Q system, Bedford, MA, USA) and DMSO were employed as reference (blank) samples. The absorbance values of the drug-Ps nanovesicles were corrected by subtracting the absorbance contributed by the blank Ps samples. Before the measurements, calibration curves were generated using pure VEM and DOX solutions ranging in concentrations from 0 to 25 mg/mL, following the methods outlined by D’Angelo et al. [31]. These calibration curves corresponded to the correlation of absorbance values with the concentrations of the respective drugs.

### 3.8. Drug Release from Chitosan Hydrogels

To examine the release of drugs from the CHs, we performed experiments using PBS buffer at pH 7.4, which mimics physiological conditions. CHs loaded with free drugs or Tf-Ps (at a concentration of 40 μg/mL) were placed in small vials with 3.0 mL of the buffer solution. These vials were then placed in a controlled environment at 37 °C with 5% CO_2_. The incubation medium was replaced every 8 h throughout the experiment to maintain physiological conditions as drugs would typically not accumulate in one region of the body. At specific time intervals ranging from 0 to 120 h, samples of the CHs were collected, lyophilized, and dissolved in 250 µL of DMSO for subsequent quantification of the drugs.

### 3.9. In Vitro Cytotoxicity Evaluation

Melanoma cells (A375, a BRAF^V600E^ malignant cell line that is sensitive to the RAF kinase inhibitor vemurafenib) and normal human umbilical vein endothelial cells (HUVECs) were seeded into 75 cm^2^ tissue culture flasks obtained from Corning Incorporated (Corning, NY, USA). The cells were cultured in RPMI 1640 medium purchased from Invitrogen (Carlsbad, CA, USA) supplemented with 10% fetal bovine serum from Hyclone (Logan, UT, USA) and 1% penicillin/streptomycin from Invitrogen. The culture medium was maintained at a pH of 7.4. The cells were cultured in a controlled environment with a humidified atmosphere at 37 °C and 5% CO_2_. To assess cell viability, the MTS assay (3-(4,5-dimethylthiazol-2-yl)-5-(3-carboxymethoxyphenyl)-2-(4-sulfophenyl)-2H-tetrazolium) from Promega Corp. (Madison, WI, USA) was employed. The cells were seeded into individual wells of a 48-well tissue culture plate at a density of 30,000 cells/cm^2^ per well. The original medium was removed after 24 h and 250 μL of fresh medium was incorporated into each well. The CHs were placed in the respective wells with nanovesicles containing varying concentrations of DOX and VEM, along with equivalent doses of the combined free drugs (not evaluated individually), as determined in Section 3.3. The control samples analyzed were empty CHs (ranging from 0.25 to 1.5 mg/mL), empty Ps (ranging from 0.05 to 0.5 mg/mL) and empty Ps incorporated into CHs (ranging from 0.05 to 0.5 mg/mL of Ps and 0.25 to 1.5 mg/mL of CHs, respectively). The plate was then stored for 3 days in a humidified atmosphere with 5% CO_2_ at 37 °C. The medium and CHs were carefully removed, and MTS reagent (250 μL) was introduced to each well. The plate was subsequently stored for a duration of 60 min. To quantify cell viability concerning the control wells (cells incubated in medium without CHs or Ps), absorbance was recorded at 490/700 nm using a F200 Infinito plate reader (Tecan System Inc., San Jose, CA, USA).

### 3.10. Analysis

Data analysis was carried out using Microsoft Excel version 16.88 (Redmond, WA, USA) and GraphPad Prism version 10.3.1 (La Jolla, CA, USA). To evaluate the effectiveness of the developed Ps nanovesicles, we employed cell viability as the primary metric in our *in vitro* cytotoxicity investigations. We collected experimental data from three independent assays and presented the results as the mean ± *SD*. Statistical significance was determined using a significance level of *p* < 0.05.

## 4. Conclusions

Our findings demonstrate the potential of using oxalate Tf-conjugated DOX+VEM PEG-PCL-based Ps incorporated within chitosan hydrogels for the targeting and treatment of malignant melanoma. This achievement involves the development of innovative biomaterials with potential anticancer properties, wherein chitosan hydrogels serve as a matrix for incorporating transferrin-conjugated nanovesicles. Using such platforms for combined chemotherapy of DOX and VEM offers numerous advantages, including ease of fabrication, cost-effectiveness, and sustained release of the drugs over 5 days. Furthermore, the surface-modified polymersomes have the potential to achieve the required therapeutic drug levels specifically at the targeted site, mitigating systemic drug exposure and reducing associated adverse reactions. These extremely encouraging results provide substantial support for the promise of this strategy as an innovative approach to advance controlled and precise drug administration employing Tf-conjugated nanovesicles in cancer treatment.

## Figures and Tables

**Table 1:** Parameters of co-encapsulation of doxorubicin (DOX) and vemurafenib (VEM) in polymersomes (Ps) composed by PEG-PCL, with and without transferrin (Tf) conjugation. *DL* and *EE* represent drug loading and encapsulation efficiencies, respectively. Error bars shown correspond to 95% confidence intervals calculated from three independent measurements.

Polymersomes	DOX		VEM	
	*DL* (%)	*EE* (%)	*DL* (%)	*EE* (%)
**Ps without Tf**	12.1 ± 3.5	35.0 ± 3.0	17.3 ± 2.5	42.2 ± 4.2
**Native Tf-Ps**	10.0 ± 2.5	32.2 ± 3.5	18.1 ± 4.0	40.4 ± 5.0
**Oxalate Tf-Ps**	11.6 ± 3.2	33.8 ± 4.5	16.3 ± 4.5	39.5 ± 4.8

## Data Availability

Data are contained within the article and Supplementary Materials.

## Supplementary Materials

The following supporting information can be downloaded at: https://www.mdpi.com/article/10.3390/ph17091177/s1, Methodologies S1.1 Transferrin conjugation onto the polymersomes [28,29] and S1.2 Generation of oxalate Tf and iron loading of Tf [28]; Figure S2: Transmission electron microscopy; and Figure S3: Western blot analysis [66].

## References

[1] Milojkovic Kerklaan B., van Tellingen O., Huitema A.D.R., Beijnen J.H., Boogerd W., Schellens JH M., Brandsma D. (2016). Strategies to target drugs to gliomas and CNS metastases of solid tumors. J. Neurol..

[2] Pratt W.B., Ruddon R.W., Ensminger W.D., Maybaum J. (1994). The Anticancer Drugs.

[3] Robbins S.L., Angell M., Kumar V. (1981). Basic Pathology.

[4] Hochberg F.H., Pruitt A. (1980). Assumptions in the radiotherapy of glioblastoma. Neurology.

[5] Jain R.K. (1994). Barriers to drug delivery in solid tumors. Sci. Am..

[6] Nawaz A., Ullah S., Alnuwaiser M.A., Rehman F.U., Selim S., Al Jaouni S.K., Farid A. (2022). Formulation and evaluation of chitosan-gelatin thermosensitive hydrogels containing 5FU-alginate nanoparticles for skin delivery. Gels.

[7] Fung L.K., Saltzman W.M. (1997). Polymeric implants for cancer chemotherapy. Adv. Drug Deliv. Rev..

[8] De Souza R., Zahedi P., Allen C.J., Piquette-Miller M. (2010). Polymeric drug delivery systems for localized cancer chemotherapy. Drug Deliv..

[9] Ho E.A., Vassileva V., Allen C., Piquette-Miller M. (2005). In vitro and in vivo characterization of a novel biocompatible polymer–lipid implant system for the sustained delivery of paclitaxel. J. Release.

[10] Brem H., Langer R. (1996). Polymer-based drug delivery to the brain. Sci. Med..

[11] DeFail A.J., Edington H.D., Matthews S., Lee W.-C.C., Marra K.G. (2006). Controlled release of bioactive doxorubicin from microspheres embedded within gelatin scaffolds. J. Biomed. Mater. Res. A.

[12] Szewczuk M., Hrynyk M., Ellis J.P., Haxho F., Allison S., Steele JA M., Abdulkhalek S., Neufeld R.J. (2015). Therapeutic designed poly (lactic-*co*-glycolic acid) cylindrical oseltamivir phosphate-loaded implants impede tumor neovascularization, growth and metastasis in mouse model of human pancreatic carcinoma. Drug. Des. Devel. Ther..

[13] Liu J., Meisner D., Kwong E., Wu X.Y., Johnston M.R. (2007). A Novel trans-lymphatic drug delivery system: Implantable gelatin sponge impregnated with PLGA–paclitaxel microspheres. Biomaterials.

[14] Zhao W., Li J., Jin K., Liu W., Qiu X., Li C. (2016). Fabrication of functional PLGA-based electrospun scaffolds and their applications in biomedical engineering. Mat. Sci. Eng. C.

[15] Elstad N.L., Fowers K.D. (2009). OncoGel (ReGel/Paclitaxel)—Clinical applications for a novel paclitaxel delivery system. Adv. Drug. Deliv. Rev..

[16] Yoon J.J., Kim J.H., Park T.G. (2003). Dexamethasone-releasing biodegradable polymer scaffolds fabricated by a gas-foaming/salt-leaching method. Biomaterials.

[17] Makadia H.K., Siegel S.J. (2011). Poly lactic-*co*-glycolic acid (PLGA) as biodegradable controlled drug delivery carrier. Polymers.

[18] Jain J.P., Modi S., Domb A.J., Kumar N. (2005). Role of polyanhydrides as localized drug carriers. J. Control Release.

[19] Daniels T.R., Bernabeu E., Rodríguez J.A., Patel S., Kozman M., Chiappetta D.A., Holler E., Ljubimova J.Y., Helguera G., Penichet M.L. (2012). The transferrin receptor and the targeted delivery of therapeutic agents against cancer. Biochim. Biophys. Acta-Gen. Subj..

[20] Wei Y., Gu X., Sun Y., Meng F., Storm G., Zhong Z. (2020). Transferrin-binding peptide functionalized polymersomes mediate targeted doxorubicin delivery to colorectal cancer in vivo. J. Control. Release.

[21] Reckhow Constance L., Enns Caroline A. (1988). Characterization of the transferrin receptor in tunicamycin-treated A431 cells. J. Biol. Chem..

[22] De Brabander M., Nuydens R., Geerts H., Hopkins C.R. (1988). Dynamic behavior of the transferrin receptor followed in living epidermoid carcinoma (A431) cells with nanovid microscopy. Cell Motil..

[23] Tao J., Liu Y.Q., Li Y., Peng J.L., Li L., Liu J., Shen X., Shen G.X., Tu Y.T. (2007). Hypoxia: Dual effect on the expression of transferrin receptor in human melanoma A375 cell line. Exp. Dermatol..

[24] Liu Y., Tao J., Li Y., Yang J., Yu Y., Wang M., Xu X., Huang C., Huang W., Dong J. (2009). Targeting hypoxia-inducible factor-1α with Tf–PEI–shRNA complex via transferrin receptor-mediated endocytosis inhibits melanoma growth. Mol. Ther..

[25] Mayle K.M., Le A.M., Kamei D.T. (2012). The intracellular trafficking pathway of transferrin. Biochim. Biophys. Acta-Gen. Subj..

[26] Yazdi P.T., Murphy R.M. (1994). Quantitative analysis of protein synthesis inhibition by transferrin-toxin conjugates. Cancer Res..

[27] Ciechanover A., Schwartz A.L., Dautry-Varsat A., Lodish H.F. (1983). Kinetics of internalization and recycling of transferrin and the transferrin receptor in a human hepatoma cell line. Effect of lysosomotropic agents. J. Biol. Chem..

[28] Lopes A.M., Chen K.Y., Kamei D.T. (2017). A Transferrin variant as the targeting ligand for polymeric nanoparticles incorporated in 3-D PLGA porous scaffolds. Mat. Sci. Eng. C..

[29] Chiu RY T., Tsuji T., Wang S.J., Wang J., Liu C.T., Kamei D.T. (2014). Improving the systemic drug delivery efficacy of nanoparticles using a transferrin variant for targeting. J. Control Release.

[30] Lao B.J., Tsai W.-L.P., Mashayekhi F., Pham E.A., Mason A.B., Kamei D.T. (2007). Inhibition of transferrin iron release increases in vitro drug carrier efficacy. J. Control Release.

[31] D’Angelo N.A., Câmara MC C., Noronha M.A., Grotto D., Chorilli M., Lourenço F.R., Rangel-Yagui C.O., Lopes A.M. (2022). Development of PEG-PCL-based polymersomes through design of experiments for co-encapsulation of vemurafenib and doxorubicin as chemotherapeutic drugs. J. Mol. Liq..

[32] Kilár F., Simon I. (1985). The Effect of iron binding on the conformation of transferrin. A small angle X-ray scattering study. Biophys. J..

[33] Grossen P., Witzigmann D., Sieber S. (2017). Huwyler JPEG-PCL-based nanomedicines: A biodegradable drug delivery system its application. J. Control Release.

[34] Jeong S.I., Kim B.-S., Lee Y.M., Ihn K.J., Kim S.H., Kim Y.H. (2004). Morphology of elastic poly(L-lactide-*co*-ε-caprolactone) copolymers and in vitro and in vivo degradation behavior of their scaffolds. Biomacromolecules.

[35] Kilàr F., Hjertén S. (1989). Separation of the human transferrin forms by carrier- free high-performance zone electrophoresis and isoelectric focusing. J. Chromatogr. A.

[36] Colley H.E., Hearnden V., Avila-Olias M., Cecchin D., Canton I., Madsen J., MacNeil S., Warren N., Hu K., McKeating J.A. (2014). Polymersome-mediated delivery of combination anticancer therapy to head and neck cancer cells: 2D and 3D in vitro evaluation. Mol. Pharm..

[37] Iatrou H., Dimas K., Gkikas M., Tsimblouli C., Sofianopoulou S. (2014). Polymersomes from polypeptide containing triblock co- and terpolymers for drug delivery against pancreatic cancer: Asymmetry of the external hydrophilic blocks. Macromol. Biosci..

[38] Salama A., Hasanin M., Hesemann P. (2020). Synthesis and antimicrobial properties of new chitosan derivatives containing guanidinium groups. Carbohydr. Polym..

[39] Abou-Yousef H., Dacrory S., Hasanin M., Saber E., Kamel S. (2021). Biocompatible hydrogel based on aldehyde-functionalized cellulose and chitosan for potential control drug release. Sustain. Chem. Pharm..

[40] Hasanin M.S., Abdelraof M., Fikry M., Shaker Y.M., Sweed A.M., Senge M.O. (2021). Development of antimicrobial laser-induced photodynamic therapy based on ethylcellulose/chitosan nanocomposite with 5, 10, 15, 20-tetrakis (m-hydroxyphenyl) porphyrin. Molecules.

[41] Wei L., Cai C., Lin J., Chen T. (2009). Dual-drug delivery system based on hydrogel/micelle composites. Biomaterials.

[42] Jaklenec A., Hinckfuss A., Bilgen B., Ciombor D.M., Aaron R., Mathiowitz E. (2008). Sequential release of bioactive IGF-I and TGF-Β1 from PLGA microsphere-based scaffolds. Biomaterials.

[43] Jindal N., Mehta S.K. (2015). Nevirapine loaded poloxamer 407/pluronic p123 mixed micelles: Optimization of formulation and in vitro evaluation. Colloids Surf. B Biointerfaces.

[44] Ulubayram K., Eroglu I., Hasirci N. (2002). Gelatin microspheres and sponges for delivery of macromolecules. J. Biomater. Appl..

[45] Meng F., Zhong Z., Feijen J. (2009). Stimuli-responsive polymersomes for programmed drug delivery. Biomacromolecules.

[46] Lee J.S., Zhou W., Meng F., Zhang D., Otto C., Feijen J. (2010). Thermosensitive hydrogel-containing polymersomes for controlled drug delivery. J. Control Release.

[47] Schmaljohann D. (2006). Thermo-and pH-responsive polymers in drug delivery. Adv. Drug Deliv. Rev..

[48] Zhou Y., Yan D., Dong W., Tian Y. (2007). Temperature-responsive phase transition of polymer vesicles: Real-time morphology observation and molecular mechanism. J. Phys. Chem. B.

[49] Qu T., Wang A., Yuan J., Gao Q. (2009). Preparation of an amphiphilic triblock copolymer with pH- and thermo-responsiveness and self-assembled micelles applied to drug release. J. Colloid. Interface Sci..

[50] Kurnik I.S., D’Angelo N.A., Mazzola P.G., Chorilli M., Kamei D.T., Pereira JF B., Vicente A.A., Lopes A.M. (2021). Polymeric micelles using cholinium-based ionic liquids for the encapsulation and release of hydrophobic drug molecules. Biomater. Sci..

[51] Salimi A., Sharif Makhmal Zadeh B., Kazemi M. (2019). Preparation and optimization of polymeric micelles as an oral drug delivery system for deferoxamine mesylate: In vitro and ex vivo studies. Res. Pharm. Sci..

[52] Castro K.C., Coco J.C., Santos É.M., Ataide J.A., Martinez R.M., Nascimento MH M., Prata J., Fonte PR M.L., Severino P., Mazzola P.G. (2023). Pluronic^®^ triblock copolymer-based nanoformulations for cancer therapy: A 10-year overview. J. Control Release.

[53] Ma H., Huang Y., Tian W., Liu J., Yan X., Ma L., Lai J. (2024). Endothelial transferrin receptor 1 contributes to thrombogenesis through cascade ferroptosis. Redox Biol..

[54] Johannessen C.M., Boehm J.S., Kim S.Y., Thomas S.R., Wardwell L., Johnson L.A., Emery C.M., Stransky N., Cogdill A.P., Barretina J. (2010). COT drives resistance to RAF inhibition through MAP kinase pathway reactivation. Nature.

[55] Singh S. (2018). Liposome encapsulation of doxorubicin and celecoxib in combination inhibits progression of human skin cancer cells. Int. J. Nanomed..

[56] Nahire R., Haldar M.K., Paul S., Ambre A.H., Meghnani V., Layek B., Katti K.S., Gange K.N., Singh J., Sarkar K. (2014). Multifunctional polymersomes for cytosolic delivery of gemcitabine and doxorubicin to cancer cells. Biomaterials.

[57] Zhou D., Fei Z., Jin L., Zhou P., Li C., Liu X., Zhao C. (2021). Dual-responsive polymersomes as anticancer drug carriers for the co-delivery of doxorubicin and paclitaxel. J. Mater. Chem. B.

[58] Curcio M., Mauro L., Naimo G.D., Amantea D., Cirillo G., Tavano L., Casaburi I., Nicoletta F.P., Alvarez-Lorenzo C., Iemma F. (2018). Facile synthesis of pH-responsive polymersomes based on lipidized PEG for intracellular co-delivery of curcumin and methotrexate. Colloids Surf. B Biointerfaces.

[59] Castro K.C., Barbosa L.R., Greaves T.L., Lopes A.M. (2024). Polymersomes-based curcumin delivery as cancer therapeutics. Curcumin-Based Nanomedicines as Cancer Therapeutics.

[60] Yang X., Hu C., Tong F., Liu R., Zhou Y., Qin L., Ouyang L., Gao H. (2019). Tumor Microenvironment-responsive dual drug dimer-loaded PEGylated bilirubin nanoparticles for improved drug delivery and enhanced immune-chemotherapy of breast cancer. Adv. Funct. Mater..

[61] Aplin A.E., Kaplan F.M., Shao Y. (2011). Mechanisms of resistance to RAF inhibitors in melanoma. J. Investig. Dermatol..

[62] Mamalis A., Garcha M., Jagdeo J. (2014). Targeting the PD-1 pathway: A promising future for the treatment of melanoma. Arch. Dermatol. Res..

[63] Nikolaou V.A., Stratigos A.J., Flaherty K.T., Tsao H. (2012). Melanoma: New insights and new therapies. J. Investig. Dermatol..

[64] Yap T.A., Omlin A., Bono J.S. (2013). Development of therapeutic combinations targeting major cancer signaling pathways. J. Clin. Oncol..

[65] Wande D.P., Qiu Y., Chen S., Yao L., Xu Y., Yao J., Xiong H. (2022). Modified chitosan nanogel-polymersomes for oral co-delivery of oxaliplatin and rapamycin for synergistic chemotherapy. J. Drug Deliv. Sci. Technol..

[66] Li S., Amat D., Peng Z., Vanni S., Raskin S., De Angulo G., Othman A.M., Graham R.M., Leblanc R.M. (2016). Transferrin conjugated nontoxic carbon dots for doxorubicin delivery to target pediatric brain tumor cells. Nanoscale..

