# Supplementary material for: Dual Chemotherapeutic Loading in Oxalate Transferrin-Conjugated Polymersomes Incorporated into Chitosan Hydrogels for Site-Specific Targeting of Melanoma Cells
Source: Pharmaceuticals (Basel). 2024 Sep 6;17(9):1177. doi: 10.3390/ph17091177 (PMC11434979; doi:10.3390/ph17091177)
Supplement: Supplementary file 1 [file pharmaceuticals-17-01177-s001.zip › pharmaceuticals-3148513-supplementary.pdf]

Supplementary Materials

Dual chemotherapeutic loading in oxalate transferrin-conjugated polymersomes incorporated into chitosan hydrogels for site-specific targeting of melanoma cells

Mariana de C. Aranha[1], Luciana M. R. Alencar[2], Eliana B. Souto[3], Daniel T. Kamei[4], André M. Lopes[1,*]

[1] Department of Biotechnology, Lorena School of Engineering, University of São Paulo (EEL/USP), Lorena, São Paulo 12612-550, Brazil.

[2] Physics Department, Laboratory of Biophysics and Nanosystems, Federal University of Maranhão, São Luís, MA, Brazil.

[3] UCD School of Chemical and Bioprocess Engineering, University College Dublin, Belfield, Dublin 4, D04 V1W8, Ireland.

[4] Department of Bioengineering, University of California, Los Angeles, 420 Westwood Plaza, 5121 Engineering V, Los Angeles, CA 90095, USA.

***Corresponding author:**

Prof. André M. Lopes

andreml@usp.br

Lorena School of Engineering

University of São Paulo

S1. Methodology

S1.1 Transferrin conjugation onto the polymersomes

In order to activate the surface carboxyl groups on the Ps, we employed the method described by Lopes et al. [28], using the 1-ethyl-3-(3-dimethylaminopropyl) carbodiimide (EDC) molecule and N-hydroxysuccinimide (NHS). Initially, 3 mg of Ps were suspended in 0.2 mL of MES buffer (100 mM, pH 5.5). To activate the carboxyl groups, we added 5 µL of EDC and 4 µL of NHS (1 mg/mL for both) to the Ps suspension and allowed them to react for 20 min. To quench the reaction, we adjusted the pH of the suspension to 7.4 by adding 0.5 M PBS buffer. For PEGylation of the Ps, we introduced heterobifunctional PEG maleimide-$PEG_{10,000}$-NH_2 (Nanocs Inc., New York, NY) at a Ps:PEG 1:5,000 molar ratio to the activated Ps suspension. The solution was then allowed to react for 3 h at 25 °C, enabling the amine groups on the heterobifunctional PEG to form a stable bond with the activated Ps. We removed any remaining free EDC/NHS and unreacted PEG by centrifugation at 9,000*g*. To couple Tf to the PEGylated Ps, we first thiolated Tf using iminothiolane (IT), following a previously described method [29]. Any excess IT was eliminated through centrifugation using Zeba desalting columns in PBS buffer. Subsequently, we added the thiolated Tf (at a 1:5,000 Ps:Tf molar ratio) to the PEGylated Ps, allowing the thiolated Tf to form a permanent link with the maleimide group on the PEGylated Ps. After incubating overnight at 25 °C, we removed any unbound Tf molecules through centrifugation at 9,000*g*. The resulting Tf-PEG-Ps (*i.e.*, Tf-Ps) were collected and stored in 50 mM HEPES buffer at 4 °C.

S1.2 Generation of oxalate Tf and iron loading of Tf

To produce both the native and oxalate versions of Tf, the iron loading procedure was varied, according to Lopes et al. [28]. Briefly, 25 µL of a 250 mM iron chelating agent (nitrilotriacetate - NTA) was combined with 15 µL of a 250 mM iron (III) chloride solution.

For the generation of the native Tf samples, the NTA and iron (III) chloride mixture was blended with a 20 mM HEPES buffer containing 20 mM bicarbonate. On the other hand, to generate the oxalate Tf samples, the NTA and iron (III) chloride mixture was instead combined with a 20 mM HEPES buffer containing 20 mM oxalate. Subsequently, this iron mixture with either bicarbonate or oxalate was added to Tf samples in a 1:110 Tf:Fe molar ratio and incubated at 25 °C for 3 h to ensure complete iron loading of all Tf molecules. Any excess free iron from the iron-loaded samples was eliminated through centrifugation at 9,000*g*.

S2. Transmission electron microscopy

The morphology of PEG-PCL polymersomes was evaluated using transmission electron microscopy (TEM, Tecnai FEI G20, Hillsboro, Oregon) at an accelerating voltage of 80 kV. For all analyses, 5 μL of each system was deposited onto 300 nm copper-coated carbon grids and stained with a 2% (w/v) phosphotungstic acid solution; excess liquid was removed using filter paper. Figures S2A and S2B show the resulting TEM images of the polymersomes.

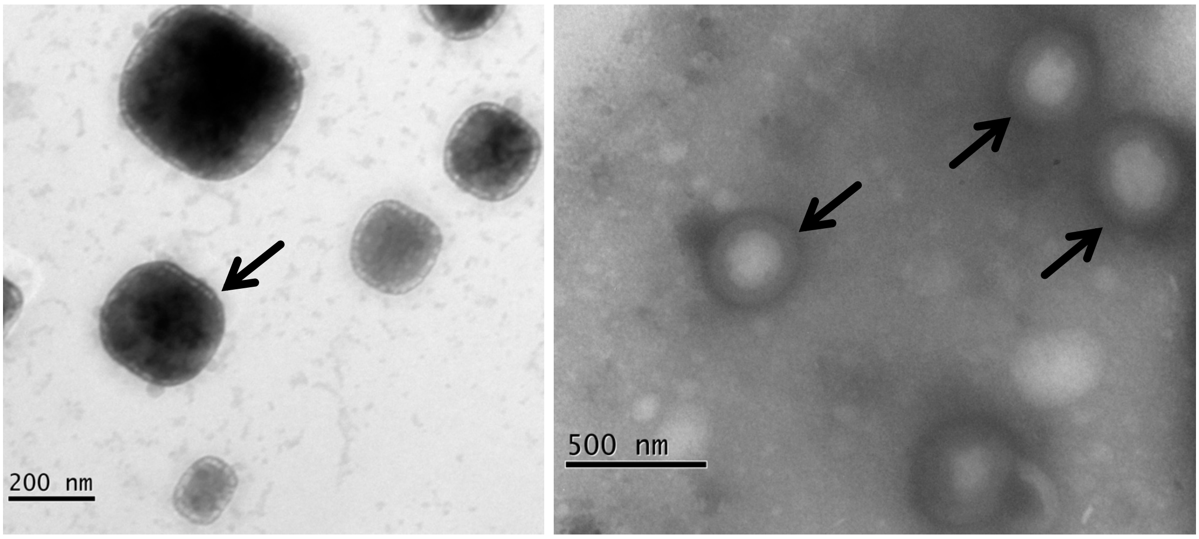

Figure S2. Transmission electron microscopy images of PEG-PCL polymersomes (**A** and **B** at different scales).

S3. Western blot analysis

Western blot analysis was performed as previously described [66]. Briefly, cells were lysed in RIPA buffer (1% NP-40 or Triton X-100, 1% sodium deoxycholate, 0.1% SDS, 150 mM NaCl, 50 mM Tris-HCl, pH 7.8, and 1 mM EDTA), and 25 µg of protein (Tf) were separated by gel electrophoresis. Proteins were transferred to nitrocellulose membranes and immunoblotted with anti-transferrin receptor 1 (TfR1) antibody (Cell Signaling Technology, Danvers, MA, USA). The results indicate that A375 cells overexpress TfR to a greater extent than HUVECs (Figure S3).

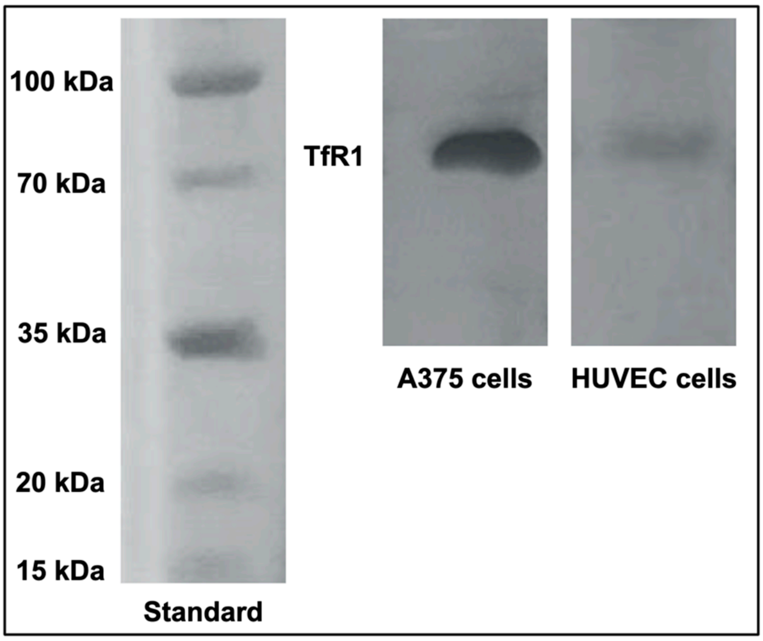

Figure S3. Characteristics of the cell lines used in this study and Western blot analysis of TfR1 levels in melanoma cells (A375, a BRAF^{V600E} mutant malignant cell line) and normal human umbilical vein endothelial cells (HUVECs).
